# LimiTT: link miRNAs to targets

**DOI:** 10.1186/s12859-016-1070-1

**Published:** 2016-05-11

**Authors:** Julia Bayer, Carsten Kuenne, Jens Preussner, Mario Looso

**Affiliations:** Group of Bioinformatics, Max Planck Institute for Heart and Lung Research, Ludwigstrasse 43, D-61231 Bad Nauheim, Germany

**Keywords:** MiRNA target interactions, miRNA target linkage, Transcriptome, Proteome, miRNAome, Niche model organisms

## Abstract

**Background:**

MicroRNAs (miRNAs) impact various biological processes within animals and plants. They complementarily bind target mRNAs, effecting a post-transcriptional negative regulation on mRNA level. The investigation of miRNA target interactions (MTIs) by high throughput screenings is challenging, as frequently used *in silico* target prediction tools are prone to emit false positives. This issue is aggravated for niche model organisms, where validated miRNAs and MTIs both have to be transferred from well described model organisms. Even though DBs exist that contain experimentally validated MTIs, they are limited in their search options and they utilize different miRNA and target identifiers.

**Results:**

The implemented pipeline LimiTT integrates four existing DBs containing experimentally validated MTIs. In contrast to other cumulative databases (DBs), LimiTT includes MTI data of 26 species. Additionally, the pipeline enables the identification and enrichment analysis of MTIs with and without species specificity based on dynamic quality criteria. Multiple tabular and graphical outputs are generated to permit the detailed assessment of results.

**Conclusion:**

Our freely available web-based pipeline LimiTT (https://bioinformatics.mpi-bn.mpg.de/) is optimized to determine MTIs with and without species specification. It links miRNAs and/or putative targets with high granularity. The integrated mapping to homologous target identifiers enables the identification of MTIs not only for standard models, but for niche model organisms as well.

**Electronic supplementary material:**

The online version of this article (doi:10.1186/s12859-016-1070-1) contains supplementary material, which is available to authorized users.

## Background

The class of microRNAs (miRNAs) consists of small, approximately 22 nucleotides long, non-coding RNAs, which play a crucial role in the negative gene regulation of many biological processes in various organisms (reviewed in [1]). Since their first discovery in the early 1990s in *Caenorhabditis elegans*, more than 28,600 miRNAs have been identified within various species [2–4]. Examples of biological processes involving miRNAs are the initiation and progression of human cancer [5, 6] or the development and disease of mammalian hearts [7, 8].

Because the negative regulation of gene expression is also induced for imperfect miRNA-mRNA seed region matches, miRNAs are able to target more than one mRNA. Consequently, mRNAs might be regulated by one or several miRNAs [9].

Considering the interpretation of biological data with respect to miRNAs, the identification of interactions between miRNAs and their target mRNAs is an essential step. Often *in silico* target prediction tools (reviewed in [10]) are used to link miRNA datasets to their targets. These tools assess sequence similarity, mRNA folding and other parameters to identify possible targets. To increase the accuracy of predictions, some tools use the characteristic properties of already validated miRNA target interactions (MTIs). Several databases like TarBase [11], miRTarBase [12], miRecords [13] and starBase [14] exist, that host these experimentally validated MTIs, mainly by curating research articles with a miRNA context (for details see below). One recently published DB that merges the information of four different resources containing validated MTIs and the data of 12 MTI prediction tools is miRWalk2.0 [15] (http://zmf.umm.uni-heidelberg.de/apps/zmf/mirwalk2/index.html). However, miRWalk2.0 is designed to work with MTIs of human, mouse and rat exclusively. The same species restriction applies for the cumulative DB miRSel [16] (https://services.bio.ifi.lmu.de/mirsel/), which combines its own validated MTI findings with the data of three other MTI DBs, as well as with computational predictions. To the best of our knowledge, no existing tool provides an option to combine and compare the data of verified target DBs of more than three species in addition to the handling of extensive lists of miRNAs or target identifiers as input, especially if these contain identifiers of various species. Due to this limitation, the search for validated MTIs becomes challenging, especially for niche model organisms without previously known miRNA repertoires (reviewed in [17]). Such organisms often host unique capabilities in certain fields of live, e.g. tissue regeneration or accelerated/delayed ageing. Examples for niche model organisms in the field of regeneration research are axolotl [18], newt [19–21], and hydra [22] due to their ability to regenerate whole extremities complex tissues and organs. By the analysis of MTIs, single miRNAs were already linked to regeneration processes of extremities and lenses within the newt, as well as the heart, limb and spinal cord of axolotls [18, 23, 24]. While there exist specialized tools that identify miRNAs from high throughput sequencing approaches in niche models (such as MIRPIPE [25]), the miRNA target assignment in such settings is still challenging. Here, miRNA and gene or protein identifiers have to be transferred from standard model organisms by homology based annotation approaches to enable a comparison with verified MTIs. The mapping onto standard organisms results in datasets containing miRNAs or genes/proteins from a variety of organisms, representing a new level of complexity. Such species overlapping datasets cannot yet be processed by any MTI DB.

Another crucial step in MTI analysis is the integration of expression data from high throughput experiments such as RNA-Seq or MS-based proteomics. For this kind of analysis, MTIs have to be evaluated in terms of their potential influence on the phenotype, allowing the identification of miRNA driven effects on gene or protein expression. The expected result is the identification of several key miRNAs that might explain the differential expression between conditions under investigation.

Here we present a user-friendly pipeline named LimiTT, intended to overcome the challenges mentioned above. LimiTT enables an automatic assignment of experimentally validated MTIs to a given set of miRNAs and possible targets (e.g. an annotated transcriptome or proteome). In order to permit application to niche model organisms, the tool is able to process species overlapping datasets which are compared to a wide range of MTIs collected from multiple MTI DBs. Furthermore, the pipeline comprises a method to consider ranked target lists to assess the potential influence of miRNAs on the phenotype under investigation by the determination of an enrichment score for miRNA target sets.

## Implementation and functionality

### Preparing the reference databases

LimiTT relies on experimentally validated MTIs originating from the open source DBs TarBase, miRTarBase, miRecords, and starBase. TarBase (http://microRNA.gr/tarbase) contains about 65,000 curated MTIs of 18 different species in its sixth version. MTIs are automatically preselected from miRNA-related PubMed [26] (http://pubmed.org) entries, manually curated and assigned to their miRBase accession numbers. This procedure is similar to the curation method of miRTarBase (http://mirtarbase.mbc.nctu.edu.tw/). The 2013 update (release 4.5) of this DB contains about 51,500 experimentally verified MTIs of 18 species. The MTI DB miRecords (release 1, update on 27.04.2013; http://c1.accurascience.com/miRecords/) contains about 2,700 MTIs of 15 species. Unlike the other two DBs, miRecords is specialised on interactions verified by Reporter assays and Western blots, whereas the others also include MTIs verified by NGS methods. Finally, StarBase (release 2.0; http://starbase.sysu.edu.cn) is focused on MTIs experimentally verified by CLIP-Seq experiments and collects interactions from three species. It retrieves its information by building an overlap of predicted MTIs processed from several miRNA prediction software programs with CLIP-Seq supported interactions from 108 data sets, generated by 37 studies. Other DBs like miR2Disease, HMDD or PhenomiR also contain experimentally validated MTIs, but just consider miRNAs connected to human diseases (reviewed in [10]).

A comparison of the selected databases mentioned above reveals a huge difference in terms of experimental methods the MTIs were validated with. Furthermore, the naming schemes of assigned targets differ, as target identifiers are acquired from curated publications, leading to varying kinds of symbols, identifiers, and accession numbers. This is obstructive not only for the comparison of the DBs, but also for the comparison with submitted target lists. LimiTT performs a pre-processing of target interaction databases to overcome these difficulties.

Since the miRNA information within the TarBase data relies on miRBase accession numbers, these accessions are mapped onto full miRNA identifiers. To enable the comparison of target symbols between the MTI DBs and to retrieve additional information for each target, all identifiers are further mapped onto UniProt accessions. Gene symbols and synonyms of all UniProtKB entries, as well as cross references to several DBs (Additional file 1, Section 1b) are compared to the target identifiers of all MTI DBs. The current combination of DBs results in 2092 miRNAs and about 570,000 MTIs for 26 species (Additional file 1, Section 2).

For local pipeline execution, all pre-processed database files are included within the download archive. The database pre-processing is performed regularly to reflect changes in underlying MTI DBs.

## Results

### LimiTT use cases

**Fig. 1:**
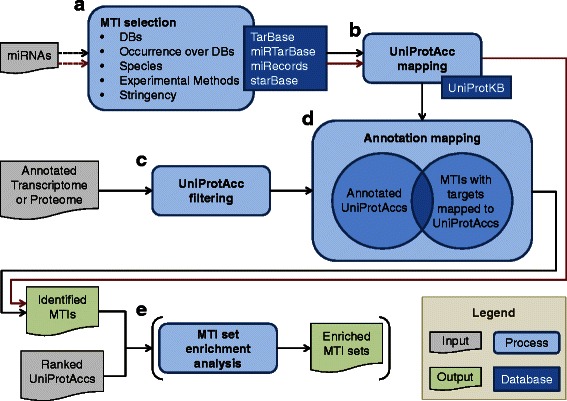
Flowchart illustrating the workflow of LimiTT. The input (grey) is composed of an optional list of miRNAs and an optional annotation file with a transcriptome/proteome. If an annotation file was submitted, the black path represents the processing steps of LimiTT, otherwise, the process is described by the red path. **a** The workflow starts with the selection of miRNA target interactions (MTIs) from the four MTI databases (DBs) in consideration of the miRNAs supplied by the user. Additionally the MTI stringency can be filtered by adjusting several parameters. **b** All target gene symbols of the selected MTIs are mapped to UniProt Accessions (UniProtAccs), while **c** all UniProtAccs are filtered from the annotation file simultaneously. **d** Subsequently, both lists are overlapped, resulting in those MTIs which can be linked to the submitted data. If no annotation file is provided, steps (**c**) and (**d**) are ignored, and the resulting MTIs rely on the miRNA list or just on the adjustable properties. **e** Optionally, an enrichment analysis of the identified MTI sets can be performed based on a ranked list with UniProtAccs supplied by the user

Depending on the input files provided by the user, LimiTT includes a range of different use cases (see Fig. 1). 1) Without any input file, the user is able to choose MTIs from the four MTI DBs by filtering them according to adjustable parameters. This mode supplies a function for comparing the DBs content for specific needs, such as filtering for MTIs of single species or reducing the DB content to MTIs with specific validation methods. 2) When submitting a list of miRNA identifiers, LimiTT generates a list of targets for each miRNA in accordance with the parameters selected for the MTI database comparison. This feature enables the identification of targets for miRNAs retrieved for example via miRNA-Seq technologies. 3) Starting the pipeline with an annotation file hosting UniProt IDs (e.g. resulting from a transcriptome screen or an annotated ChIP-Seq analysis) will filter for validated MTIs with targets present in this file. The result list will include all miRNAs that are relevant for the provided targets. 4) In case a miRNA list and a target file are submitted, the resulting MTIs will rely on both datasets. This setup will identify and link MTIs from a submitted miRNA-Seq and e.g. a ChIP-Seq or RNA-Seq. 5) Optionally, the MTI set enrichment analysis (MTISEA) function of LimiTT can be used by submitting a ranked target list to LimiTT. The origin of ranking is arbitrary and might originate from a network analysis or an expression screening e.g. from a proteomics study (see section validation below). For all use cases the provided lists can be species specific or species overlapping. A comparison on the unique features of LimiTT can be found in Additional file 2.

### General workflow

The default workflow of LimiTT starts with the input of a list of miRNAs and a file containing targets with their corresponding UniProt accessions (Fig. 1). Additional information about the file formats with examples can be found in Additional file 1.

Supplied miRNAs (as generated by an analysis tool such as MIRPIPE) are compared via a semantic comparison to the miRNA identifiers of experimentally validated MTIs from up to four MTI DBs based on user selection. The pipeline is able to process full miRNA identifiers (e.g. hsa-miR-301b-3p), as well as shortened miRNA identifiers without species prefix and/or omitted-3p, -5p suffix (e.g. miR-301b or miR-301b-3p) (Additional file 1, Section 4ab). Further options include the degree of conservation in the MTI DBs (e.g. present in one DB, or present in all DBs), the species of interest (or ignoring the underlying species), a filter for the experimental methods, as well as the stringency in case of starBase (the minimal number of CLIP-Seq experiments the MTIs are supported with) (Additional file 1, Section 4).

All MTIs fitting to the selected parameters are filtered from the chosen MTI DBs and saved separately for each chosen MTI DB. Target symbols of the selected MTIs are then mapped onto UniProt accession to facilitate the comparison between MTI DBs. At this point, the species information can be used to either map the target in a species specific way (e.g. human *hprt1* results in two UniProt accessions), or species membership can be ignored and target symbols are mapped regardless of the underlying species (e.g. *hprt1* results in 75 accessions from 56 different species). This feature permits the inclusion of homologous genes in the MTI identification.

Next, all UniProt accessions from the MTI list as well as those from the submitted target list are overlapped to identify all accessions which represent both experimentally validated miRNA targets and annotated components of the organism under investigation.

### MTI set enrichment analysis (MTISEA)

With the resulting sets of MTIs an enrichment analysis can be performed by passing an expression file containing a ranking value for each potential target (Additional file 1: Table S3). The implemented enrichment analysis is a variant of GSEA [27]. Briefly, with a running sum statistic, a weighted Enrichment Score (ES) is calculated for each gene set based on position dependent gene matches between the ranked list and the set. A leading edge analysis [27] additionally identifies and analyses the core genes of the gene set which mainly affect the ES. To take the set sizes into account, MTI set enrichment analysis calculates the Normalized Enrichment Score (NES) [27] for each gene set by using permutations (number of permutation can be set as a parameter; random permutations are performed on target genes in each miRNA set, keeping the number of targets in the respective miRNA set constant) of the dataset in the next step. Additionally, the False Discovery Rate (FDR) q-value is calculated [27], representing the estimated probability of a false positive result for each set with a given NES. MTISEA is fully integrated into LimiTT.

### Output

**Fig. 2:**
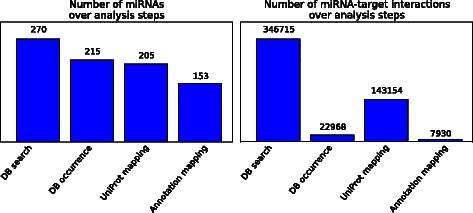
Bar graph output of LimiTT. The figure depicts an exemplary bar graph output of LimiTT, showing the number of miRNAs on the left and the number of MTIs on the right after specific processing steps of the pipeline

The pipeline generates a set of result files and figures (an example of each output file can be found in Additional file 1), each of which focuses on a specific point of view on the data. To give a general overview on the processed data, a bar graph displaying the number of miRNAs and MTIs after each processing step of the pipeline is generated (Fig. 2). These plots are very helpful for reference database depending parameter fine tuning. A MTI matrix file constitutes all interactions between identified miRNAs and target UniProtAccs (Additional file 1: Table S2). By using binary strings within the matrix, the individual occurrence of each MTI over the chosen MTI DBs is represented. In order to display all interacting miRNAs for a specific target UniProtAcc, the MTI information file lists the target gene symbols and synonyms, the corresponding UniProt accession, the species, as well as further information like protein names and GO numbers.

**Fig. 3:**
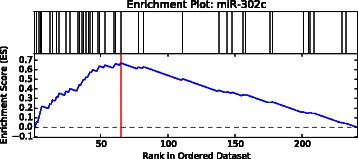
Enrichment plot output of LimiTT. Depicted is the example of an Enrichment plot for an MTI set named miR-149 from LimiTT with the running enrichment score for each of the UniProt accessions from a ranked list in blue, the positions of targets from the MTI set within the ranked dataset in black and the position of the maximum ES in red

The overlap of targets between all identified MTI sets is depicted in an MTI set overlap heatmap (Additional file 1: Figure S4). In case a MTISEA is performed, just the leading edge genes of all MTI sets are considered. After the optional MTISEA, the ranking file contains the results of the analysis for each set of miRNA targets (e.g. set size, ES, NES and FDR q-value). If no MTISEA was started, the MTI sets are ranked according to their number of targets. In case of a MTISEA, additional enrichment plots are created which illustrate the running enrichment score for each MTI set over all UniProtAccs in the ranked dataset (Fig. 3). Finally, the MTI set target information file of LimiTT represents the textual base for all enrichment plots, listing the index in the ranked list for each ranked MTI, as well as the running ES and the leading edge status.

### Validation

In order to test our pipeline for datasets from well annotated organisms as well as niche model organisms, we performed an exemplary analysis on a disease in humans, where the role of miRNAs is well described. In a second analysis we simulated a niche model dataset based on human genes and target interactions to illustrate the principle of gene identifier and miRNA target assignments in niche models.

### Testcase well annotated organism

In 2014, Bertero et al. [28] published a paper describing the identification of human MTIs relevant for pulmonary hypertension (PH) based on a sophisticated bioinformatics approach. We chose this disease model to validate LimiTT, since the role of many miRNAs is already described for PH (reviewed in [29–32]), allowing the final validation of the resulting list generated by LimiTT.

Bertero et al. used *in silico* predicted MTIs generated with a list of 242 human PH-related genes (from literature search) and created a network revealing the underlying connections among the MTIs and among the genes. Based on MTI set size and network position (highly connected knots vs. less connected), the group narrowed their findings down to 30 top ranked miRNA families consisting of 98 miRNA members (no distinction between -3p/-5p variants).

To test whether LimiTT is also able to identify at least these 30 published miRNA families just by mapping to overlapped target databases without a bioinformatics network approach, the 242 PH-related genes were used as annotation file input. LimiTT was invoked from the web interface with the following parameters: Clustered miRNAs (-3p and -5p suffixes are ignored), all MTI databases, MTI database occurrence of at least one, organism *H. sapiens*, all experimental methods and starBase stringency of one.

The pipeline identified 222 of the 242 genes as validated miRNA targets, interacting with various miRNAs. All of the top 30 miRNA families published in Bertero et al. (2014) were identified by our tool and consisted in total of 73 of the 98 miRNA members (Additional file 1: Table S5). In addition, 317 further interesting miRNAs were determined by our tool, resulting in 390 identified miRNAs in total.

Next, we tested whether our MTISEA module was able to sort the 390 identified miRNAs in a way that pulmonary hypertension (PH) relevant miRNAs will receive a high score. LimiTT was started again with the previously described annotation file consisting of the 242 PH-related genes accompanied by a ranking file. The latter contained the genes of the PH-network from Bertero et al. ranked by the number of connections to other genes within the network. The idea behind this approach is to identify miRNAs that affect highly interconnected targets within the PH-network. The resulting list was filtered for small target sets and ranked according to the normalized enrichment score (NES) calculated by our tool.

Twenty-three miRNAs of the top 25 identified sets can be assigned to the 14 miRNA families, namely mir-22, mir-28, mir-34, mir-155, mir-185, mir-193, mir-302, mir-302_2, mir-320, mir-368, mir-432, mir-515, mir-542 and mir-708 (Additional file 1: Table S6). According to miRBase, the other two miRNAs (miR-4306 and miR-3619) are not yet assigned to any family.

Fifteen of the 25 miRNAs from 10 miRNA families are already associated with pulmonary hypertension: miR-193a and miR-193b, as well as miR-22 were found to be significantly downregulated in the case of PH [33, 34], while miR-34a seems to be higher expressed [35]. Additionally, the knockout of miR-155 was found to prevent fibroblast proliferation in hypoxic conditions [36].

The miRNAs miR-302a/b/c/d of the mir-302 family were identified to be downregulated by bone morphogenetic protein (BMP) signalling, which leads to the de-repression of their target gene BMPR2 [37]. The BMP signalling pathway in turn is well known to cause heritable PH in the case of mutational defects in BMPR2 [38].

In case of a hypoxia-induced PH, the miRNA processing endonuclease Dicer is known to be downregulated, resulting in a decreasing level of miR-185 and other miRNAs [39]. This leads to the de-repression of the miR-185 target gene HIF-2, which is involved in the regulation of hypoxic adaptions in pulmonary vasculature.

The last five miRNAs can be associated with diseases that can lead to PH:

PH is known to seriously complicate idiopathic pulmonary fibrosis (IPF) [40, 41]. MiR-326 and miR-542 were found to be downregulated in pulmonary fibrosis, dysregulating homeostasis of the lung [42, 43].

The miRNAs miR-376a/b of the mir-368 family are significantly downregulated in case of patients with sickle cell disease [44]. This disease is complicated by PH in about 30 % of the SCD cases [45–47].

PH can also be caused by pulmonary tuberculosis [48, 49]. MicroRNA miR-432 was found to be to be significantly upregulated in case of tuberculosis and considered to be a biomarker for this disease [50].

Another six miRNAs of 3 miRNA families cannot be associated directly with PH, but are known to play a role in lung cancer.

The miRNAs miR-320 and miR-708 were found to be overexpressed [51, 52] in case of lung cancer, whereby a downregulation was reported for miRNAs of the miR-515 family [53, 54].

Summing up, our test dataset from Bertero et al. [28] consisting of PH-relevant genes revealed a list of miRNAs that was also reported by Bertero to be highly important in the PH disease condition. Whereas Bertero et al. reported miRNA families, our tool is also able to report single miRNA family members. Additionally, the LimiTT analysis of the PH-related gene network generated by Bertero et al. gave rise to a list of enriched MTI sets which led to miRNAs that are already well known to play a role in the PH disease.

### Testcase niche model organism

**Fig. 4:**
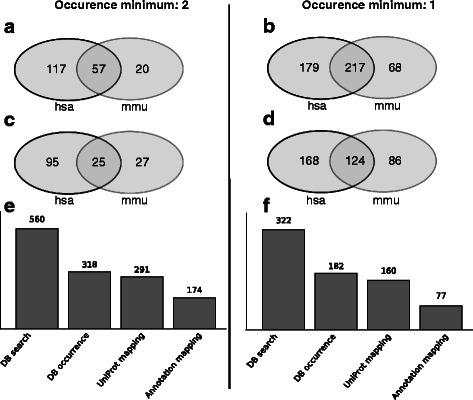
Simulated niche model dataset. Depicted is the overlap on identified miRNAs and significantly enriched miRNAs in respect to a simulated dataset where we treaded a human identifier list in context of the mouse organism. **a** Overlap of mouse and human miRNAs, identified in at least two databases. **b** Overlap of mouse and human miRNAs, identified in at least one database. **c** Overlap of significantly enriched mouse and human miRNAs, identified in at least two databases. **d** Overlap of significantly enriched mouse and human miRNAs, identified in at least one databases. **e** Total number of human miRNAs found in all four databases and respective analysis steps resulting in 174 relevant miRNAs as shown in Fig. 4a (117 + 57 = 174). **f** Total number of mouse miRNAs found in all four databases and respective analysis steps resulting in 77 relevant miRNAs as shown in Fig. 4a (57 + 20 = 77)

As MTI databases lack information about niche model organisms, a benchmark with an outcome that can be interpreted in terms of correctness is difficult to define. Therefore we simulated a niche model dataset by generating an example list of human gene symbols to serve as a ranked list for MTISEA analysis (random 1600 gene symbols). Next we assumed the human organism as niche model. In order to analyse this dataset, we choose the mouse as a well-represented and sufficiently related organism to perform a simulated annotation step. We mapped the human gene symbols to mouse uniprot identifiers, assigning 5812 mouse uniprot IDs to the original 1600 human genes in the ranked list. This step simulates the mapping step to mouse uniprot identifiers normally done by sequence homology based annotation methods as regularly performed for a real niche model organism. Next, we evoked LimiTT with the translated mouse identifiers and generated the standard output which represents the niche model results. In order to evaluate the findings, we mapped the original human gene list to human uniprot identifiers as well (7699 uniprot ids). A second run of the LimiTT tool with this ranked list represented the “real” result of the simulated human niche model for a target-performance comparison. First we compared the miRNAs that were identified in mouse and human analysis to check if miRNA targets are conserved in general on a random gene list. As shown in Fig. 4a, the miRNA identification step resulted in a larger number of miRNAs in humans compared to mouse. Nonetheless, the percentage of overlap (74 %, Fig. 4a) with identified mouse miRNAs suggests substantial conservation, considering that ~54 % of all human miRNAs are human or at least primate specific [55]. We assume the differences in the total number of detected miRNAs for human and mouse identifiers to be generated by the total difference of miRNA targets represented in the MTI databases, as shown by Fig. 4e and f. To check for robust results with respect to the MTI database settings, we performed a second run with our tool, taking targets into account that occur only in one of the MTI database. This analysis resulted in a similar overlap for mouse and human (77 %, Fig. 4b) with higher total numbers of detected miRNAs, supporting our method to be robust with respect to database composition.

Finally, we aimed to determine whether the high overlap between our simulated niche model and the real target organism is conserved for significantly enriched miRNAs from MTISEA as well. By overlapping the significantly enriched miRNAs (MTISEA FDR < 0.05), we found 50 % (Fig. 4c) to 59 % (Fig. 4d) of the individual miRNAs to be conserved (Additional file 3). In summary, the simulated niche model dataset illustrates a meaningful application of LimiTT for an organism without descriptions on the miRNA level.

## Discussion

The identification and validation of MTIs is still a challenging process, as it is not yet possible to retrieve such interactions automatically via high-throughput technologies. LimiTT offers access to experimentally validated MTIs of 26 different species by combining research from TarBase 6.0, miRTarBase 4.5, miRecords 1.0, and starBase 2.0. These particular DBs were chosen because all of them host MTIs of more than one species, their last update was in 2013 or later, and their content is publicly available.

While tools such as miRWalk2.0 and miRSel already represent such cumulative DB search tools, they are limited to MTIs of the species human, mouse, and rat. The ability to map data in a species-agnostic way represents a unique feature of LimiTT that can be used for the predictive identification of related targets based on homology to the validated ones. Extensive lists of species overlapping miRNAs and/or targets can be submitted, from which validated MTIs are filtered. This is an essential feature for the processing of niche model organisms, based on previous research finding miRNA seed conservation among mammals, particularly in the 3′ UTR, and indicating selective evolutionary pressure to maintain nucleotide binding sites for microRNAs [56, 57]. Another study on 10 mammalian orders found a whole class of MTIs to be under evolutionary constrains [58]. Due to the increasing divergence of MTIs with increasing evolutionary distance of the compared organisms, homology based mapping will nonetheless introduce a certain amount of inaccuracy [59]. Our simulated dataset suggested that an inter-species mapping results in a valid miRNA identification rate around 70 %, which reflects at least the most conserved miRNAs between species under investigation represented in the dataset. Although the evolutionary distance from human to mouse is relatively large, a substantial number of significantly enriched miRNAs (~50-60 %) could be detected. A homology based approach as implemented by LimiTT thus represents an option to bioinformatically enable research on new niche model organisms besides mouse, human and zebrafish.

Submitted miRNA identifiers can either include the species prefix and the -3p or -5p hairpin-arm information (e.g. hsa-miR-1a-3p), but can also be unspecific with regard to these information (e.g. miR-1a-3p, hsa-miR-1a or miR-1a). This function enables the clustering of the assigned miRNAs under shortened, more general identifiers submitted by the user, thus granting additional control over the sensitivity/specificity of the algorithm.

Finally, the MTI set enrichment analysis enables the combination of the miRNA target assignment with an automated functional downstream analysis which allows the identification of e.g. MTI sets whose targets show particularly high expression values. Other ranking values, such as the number of interactions between genes, can also be used for the enrichment analysis, as demonstrated in the validation section.

One current shortcoming of LimiTT is the reliance on gene symbols (gene names) to identify homologous genes, which could be improved by additionally assessing the protein similarity to exclude genes which bear the same name but putatively perform different functions.

## Conclusions

In summary, LimiTT is the first web-based pipeline which can automatically identify and link validated MTIs from extensive lists of miRNAs and target genes in batch mode, even if the provided data is not species specific. The latter enables the prediction of homologous targets for identified MTIs, extending the usability from standard model systems to niche model organisms. Furthermore, it permits the upload of ranked expression lists of miRNA effectors originating from e.g. microarrays, RNA-Seq, or proteomics experiments, which can be used to predict key miRNAs responsible for the phenotype of a dataset. The wide range of parameters permits individual filtering of the DBs in accordance with the researcher’s needs and completes the pipeline. The generated output files display different points of view on the total dataset, allowing further downstream analysis without the need to rearrange and recalculate single lists. LimiTT thus represents a valuable new tool to rapidly scan large amounts of data from high throughput research to identify miRNA/target interactions without large investments in on-site computational hardware.

## Availability and requirements

- **Project name:** LimiTT
- **Project home page:**https://bioinformatics.mpi-bn.mpg.de
- **Operating system(s):** Platform independent
- **Programming language:** Python
- **License:** Free
- **Any restrictions to use by non-academics:** None

### Availability of data and materials

All materials used for pipeline evaluation are included as supplemental files. Example files are available from our website.

## Additional files

Additional file 1: Data: Supplementary figures and descriptions helpful for the understanding of LimiTT. (PDF 1745 kb) Additional file 2: Data: Supplementary table, comparing MTI databases and other MTI tools with LimiTT (XLSX 10 kb) Additional file 3: Data: Supplementary table, result list from LimiTT MTISEA analysis on random list of identifiers as used in use case two. Each tab contains all miRNAs identified in human and mouse respectively with the parameter “database overlap (occ)” one and two. Yellow background indicates the significant enriched (FDR < 0.05) miRNAs. (XLSX 63 kb)

## Abbreviations

DB: database
GSEA: gene set enrichment analysis
miRNA: microRNA
MTI: miRNA target interaction
MTISEA: MTI set enrichment analysis
