# Supplementary material for: LimiTT: link miRNAs to targets
Source: BMC Bioinformatics. 2016 May 11;17:210. doi: 10.1186/s12859-016-1070-1 (PMC4866021; doi:10.1186/s12859-016-1070-1)

LimiTT Additional File 1

Contents

1. Pre-processing ... 2
   a. MTI database data ... 2
   b. UniProtKB data ... 2
2. MTI Database combination Version 0.1 ... 3
3. Input ... 4
   a. Annotation File ... 4
   b. miRNA File ... 5
   c. Ranking File ... 6
4. Parameter Selection ... 7
   a. miRNAs ... 7
   b. MTI databases and occurrence ... 7
   c. Species ... 8
   d. Experimental methods ... 8
   e. Stringency ... 8
5. Output ... 8
   a. Bar Graphs ... 8
   b. MTI matrix ... 9
   c. MTI information file ... 9
   d. MTI set overlap Heatmap ... 10
   e. Ranked MTI sets file ... 10
   f. Enrichment plots ... 11
   g. MTI set gene file ... 11
6. Validation ... 13
7. References ... 15
8. Manual ... 16

Contents ... 16
HowTo: Load example data ... 17
HowTo: miRNA identification ... 18
HowTo: miRNA enrichment analysis ... 19
HowTo: miRNA target search ... 20
HowTo: miRNA target identification in respect to an gene profile ... 21
HowTo: miRNA target identification in respect to an expression profile ... 22
HowTo: Inspect and download result tables/figures ... 23

1. Pre-processing

a. MTI database data

The contents of all four MTI DBs (TarBase [1], miRTarBase [2], miRecords [3] and starBase [4]) were downloaded and pre-processed to easily access and compare the MTIs. Because the miRNA information within the TarBase data solely consisted of miRBase [5] accession numbers, a method was implemented to map the accessions onto full miRNA identifiers. Therefore, a flatfile from miRBase containing the required information was downloaded and converted to a python dictionary with miRBase miRNA accessions as keys and the corresponding miRNA identifier as values to convert miRBase accession numbers at runtime. For local execution, all pre-processed database files are included within the download archive. The database pre-processing is performed regularly.

After the combination of all MTIs originating from the four MTI DBs, all species abbreviations were assigned to their full scientific names and vice versa, and the assignments were saved within a dictionary. Another dictionary was created for assigning the species to the categories Animals, Plants, Fungi, Protozoa and Viruses and vice versa. A third dictionary is required for the experimental methods: While TarBase uses consistent categories of experimental methods for its MTIs, miRTarBase and miRecords use a wide range of different terms for the methods, probably adopted from the original source. Because this data inconsistency inhibits an acceptable identification of MTIs validated by a specific experimental method, each method was assigned to one of the six categories Reporter assay (e.g. GFP and luciferase assay), Western blot, qPCR (qPCR and RT PCR), Microarray, Next generation sequencing (NGS; e.g. ChIP-Seq, CLASH and Degradome) and Other (e.g. 5'-Race, ELISA, Northern blot and Proteomics). These three dictionaries are either used for the mapping of gene symbols onto UniProtAccs, or for enabling a selection of MTIs concerning species and experimental methods.

Because each MTI DB update could result in a change of at least one of the three dictionaries, a script compares the dictionary's content with the updated datasets and initiates assignment of data not existent within the dictionaries.

b. UniProtKB data

All available data from UniProtKB [6] was downloaded in text format and pre-processed to retrieve a trimmed list of reviewed (Swiss-Prot) and unreviewed (TrEMBL) UniProtKB entries, including information about UniProt accessions, species, gene/protein names and synonyms, cross-references to the database RefSeq [7], UniGene [8], Ensembl [9], GeneID [10] and KEGG [11]. Subsequently a database-like structure with three dictionaries was created, containing the trimmed information limited to UniProtKB entries that could be associated with symbols from the MTI DBs and that possess a UniProtAcc. The first dictionary contains unique identifier numbers linked to each of the 2,170,850 trimmed entries. In the second dictionary all UniProtAccs are listed with their corresponding entry number. The third dictionary is filled with all 21,433 gene names, synonyms and cross-reference

symbols, linked to a list of the corresponding entry numbers. This procedure enables easy and fast access to information about each UniProtAcc and each stored symbol during the processing of the pipeline.

2. MTI Database combination Version 0.1

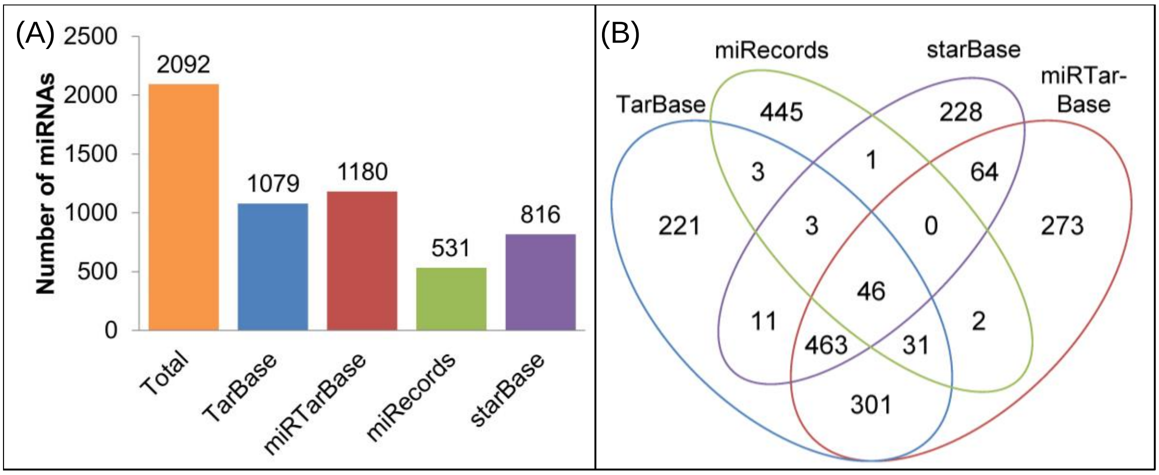

Figure 1: Number of miRNAs per MTI database
Bar graph (A) depicts the number of miRNAs for the miRNA target interaction (MTI) databases TarBase (blue), miRTarBase (red), miRecords (green) and starBase (purple) together with the total number of miRNAs over all databases (orange). Venn diagram (B) represents the number of overlapping miRNAs in the four MTI databases.

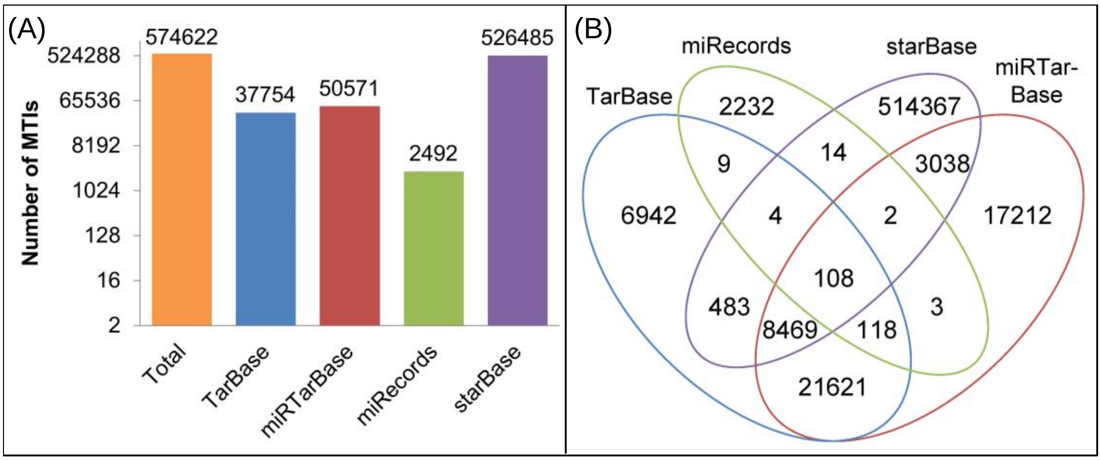

Figure 2: Number of MTIs per MTI database
(A) depicts the number of miRNA target interactions (MTIs) for the MTI databases TarBase (blue), miRTarBase (red), miRecords (green) and starBase (purple) together with the total number of MTIs over all databases (orange). The y-axis (Number of MTIs) is in log2 scale with each mark signifying an eightfold increase of MTIs starting from two. (B) represents the number of overlapping MTIs over the four MTI databases.

Tabel 1: Species within LimiTT
Table with the number of miRNA target interactions per species and per MTI DB

Category	Species	TarBase	miRTarBase	miRecords	starBase
Animals	Homo Sapiens	22818	37460	1707	423405
	Mus musculus	9371	9218	397	90205
	Caenorhabditis elegans	3796	3209	22	12875
	Rattus norvegicus	433	302	160	0
	Drosophila melanogaster	224	122	101	0
	Danio rerio	143	107	59	0
	Gallus gallus	21	22	21	0
	Bos taurus	8	7	8	0
	Bombyx mori	4	2	2	0
	Xenopus laevis	5	1	4	0
	Ovis aries	3	2	0	0
	Xenopus tropicalis	0	1	2	0
	Oryzias latipes	0	1	0	0
	Ciona intestinalis	0	0	1	0
Protozoa	Trichomonas vaginalis	0	0	1	0
Fungi	Candida glabrata	0	0	1	0
Plants	Arabidopsis thaliana	116	92	0	0
	Medicago truncatula	302	0	0	0
	Oryza sativa	106	0	0	0
	Physcomitrella patens	319	0	0	0
	Glycine max	48	0	0	0
	Vitis vinifera	10	0	0	0
Viruses	Kaposi sarcoma-associated herpesvirus	19	17	4	0
	Epstein Barr virus	6	6	0	0
	Human cytomegalovirus	2	1	0	0
	Mareks disease virus	0	1	0	0

3. Input

All input files (miRNA file, annotated transcriptome/proteome, gene/protein expression file) can either be uploaded using our Upload Tool on the website (Helpful Tools / Get Data / Upload Files) or using an account on our FTP server. The latter is only possible after user registration, which automatically creates an account with the same username (=email) and password on the FTP server (ftp://bioinformatics.mpi-bn.mpg.de/). After uploading to the FTP the files have to be uploaded to the server (Helpful Tools / Get Data / Upload Files). The data will be deleted from the server after two weeks. The Website allows user identification, which provides workspaces that can be reused without uploading files again after return to the webpage.

a. Annotation File

File Type: Tab delimited

Header: No

Required content: UniProt accessions per line or separated by comma.

Allowed content: Several columns, empty content and accessions with attached information concerning for example the underlying database, delimited by a pipe (|) symbol (e.g. sp|Q9XS59|S6A15_BOVIN). At this, only this identifier will be saved, which occurs after the first pipe symbol (e.g. sp|Q9XS59|S6A15_BOVIN > Q9XS59). Identifiers from other databases are ignored.

File Examples "Required":

A2A6A1
O88898
G5E870
Q6A037

or

A2A6A1,P54763,Q3UHC0,O88898

File Example "Allowed":

comp1000228_c0_seq1		
comp1000309_c0_seq1	slc15a3	Q8IY34,O75618
comp1000318_c0_seq1		
comp1000627_c0_seq1	slc6a15	sp|Q9XS59|S6A15_BOVIN
comp1000899_c0_seq1		gb|CX212397.1,dbj|DB530926.2,gi|154363325

The user has to define the column which includes the UniProt accessions, and it is optional to choose one column with additional information (e.g. column 1 for the transcript identifiers) and a description of this information (e.g. "Transcript") which will be included in the output of LimiTT.

Parameters:

Parameter	Description	Default
File	The uploaded annotation file	-
Column of UniProt Accessions	Number of the column which contains UniProt accessions.	3
Column of additional information	Number of the column with additional information to save for the corresponding UniProt accession.	1
Description of additional information	A keyword describing the additional information.	Transcript

b. miRNA File

File Type: Tab delimited
Header: No
Required content: One mature miRNA identifier (e.g. hsa-miR-17a-5p) per line in column 1.
Allowed content: Several columns, and shortened miRNA identifiers. Shortened miRNA identifiers have to consist at least of the prefix miR, lin or let, the identification number and, if existent, the lettered suffix showing sequence similarity (e.g miR-17a).

File Example "Required":

miR-93b
miR-36f
miR-29d
miR-29c

File Example "Allowed":

The example is a part of an original output of the MIRPIPE pipeline [12], which the parameters are adjusted to.

miR-93b	52	1.00	170	CAAGTGCTGTTCGTGCAGGTAG	33
miR-36f	211	0.00	171	ATTGAGCTATCTGTGTAG	211
miR-29d	141233	0.02	172	TAGCACCATATGAAATCAGTGT	133582
miR-29c	55690	1.00	172	TAGCACCATTTGAAATCGGTTA	44200

MiRNA identifiers have to be listed in the first column. Additionally it is possible to choose one column with additional information (e.g. column 5 for the miRNA sequences) and a description of this information (e.g. "miRNA sequence") which will be included in the output of LimiTT.

Parameters:

**Parameter**	**Description**	**Default**
File	The uploaded miRNA file	-
Column of additional information	Number of the column with additional information to save for the corresponding miRNA.	5
Description of additional information	A keyword describing the additional information.	miRNA sequence

c. Ranking File

File Type: Tab delimited

Header: No

Required content: UniProt accessions in column one, corresponding ranking value in column 2.

Allowed content: The content must not be sorted by the ranking values.

File Example:

A2A6A1	0.152108244
P54763	0.640846805
Q3UHC0	0.931454837
O88898	0.240325584
G5E870	0.47554716
Q6A037	0.495874819

The file is used for the MTI set enrichment analysis (MTISEA). Content is automatically sorted by the values in ascending order. During the analysis the Enrichment Score (ES) for each MTI set is

calculated, for which the user is allowed to choose the weighting of ranking values, which will influence the calculation. Additionally the user can decide how many simulated MTI sets are used to obtain the normalization of the calculated ES and a False Discovery Rate (FDR) of this Normalized Enrichment Score (NES).

Parameters:

Parameter	Description	Default
File	The uploaded ranking file	-
Weighting	The weighting of the ranking values to calculate the ES per MTI set.	Normal (1)
Number of permutations	The number of permutations to calculate the Normalized Enrichment Score (NES) per MTI Set.	1000

4. Parameter Selection

a. miRNAs

For each miRNA listed in the optional miRNA file, MTIs with an appropriate miRNA notation are selected. It is not just possible to choose miRNAs by their full identifiers, but also by shortened identifiers which need to consist solely of the prefix miR, lin or let, the number and, if existent, the lettered suffix showing sequence similarity. As a result, all miRNAs matching with this core identifier are clustered under this shortened name, ignoring species and hairpin arm information. Passing for example miR-123a to the pipeline, LimiTT will group all MTIs with miRNA identifiers like miR-123a-5p, miR-123a-3p and miR 123a* of all species under the passed miRNA identifier. If no lettered suffix is given (e.g. miR-123), LimiTT again just clusters miRNAs with the additional suffixes -5p, -3p or * (e.g. miR-123-5p, miR-123-3p, miR-123*).

The possibility to cluster miRNAs under their shortened identifiers is also possible if no list of miRNAs was passed to the pipeline.

b. MTI databases and occurrence

By default, LimiTT uses MTIs from all four MTI DBs TarBase, miRTarBase, miRecords and starBase. However, it is possible to use the information just of some of the DBs and ignore others. If more than one DB was selected, the parameter “Occurrence over DBs” can be used to define the minimum number of DBs the MTIs have to occur in. While the default value is set to two DBs, it is automatically changed to the number of selected DBs in the case of a manually defined value higher than the number of chosen MTI DBs.

c. Species

LimiTT offers the possibility to either use MTIs of all species, which is the default, or to filter MTIs by choosing single species or one or more of the five species categories Animal, Plant, Fungus, Virus and Excavate. The third possibility is to ignore the species of each MTI, which has a special impact on the mapping of target gene symbols onto UniProtAccs (see **Error! Reference source not found.**).

d. Experimental methods

MTIs can be selected by the experimental methods they were validated with. LimiTT provides the selectable method groups Reporter assay, Western blot, qPCR, Microarray, NGS and Other. Each group consist of several keywords suitable for the corresponding method , which will be compared to the methods saved for the MTIs. Just the command line based version of LimiTT additionally enables to filter MTIs by user-defined keywords for experimental validation methods.

e. Stringency

If the MTI DB starBase was chosen, the MTIs from this DB can be filtered by the minimal number of CLIP Seq experiments they are supported with. The corresponding parameter is named stringency and has an effect solely on the MTIs filtering for starBase.

After all MTIs fitting to the selected parameters are filtered from the chosen MTI DBs, the MTIs which occur at least in the defined number of DBs are saved internally and separately for each chosen MTI DB.

---

5. Output

a. Bar Graphs

The bar graphs (***Figure* 3**) provide an example overview of the number of miRNAs and MTIs after the different processing steps of LimiTT. MiRNAs and MTIs are counted after searching the MTI databases, after filtering by their occurrence over the DBs, after mapping MTIs onto UniProtAccs and after mapping the remaining MTI targets onto the annotated UniProtAccs. Thus, the last number within the bars is the final result.

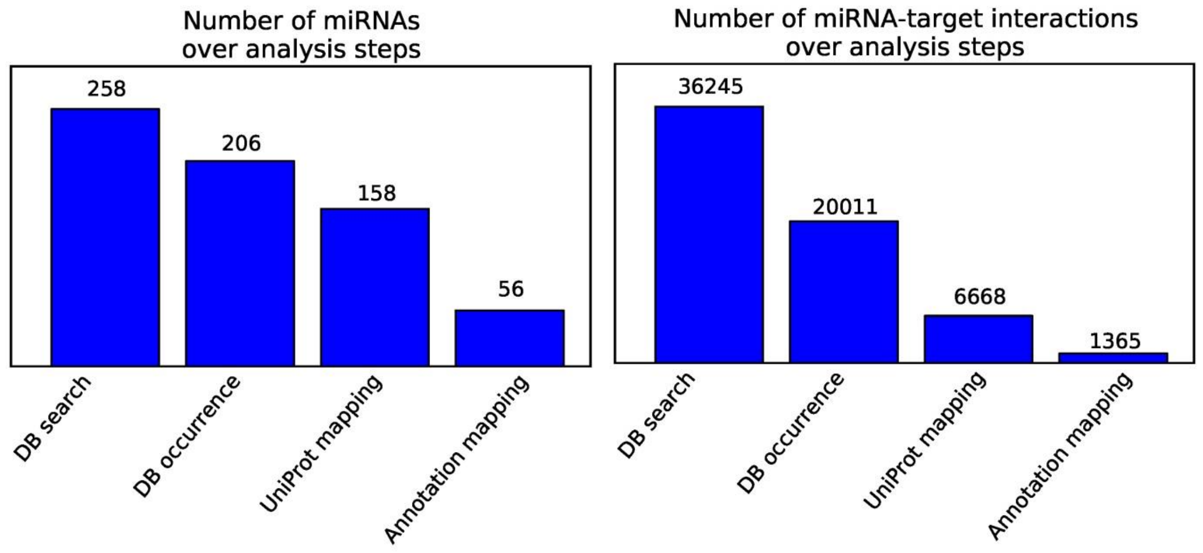

Figure 3: Bar Graph output of LimiTT
The figure depicts an exemplary bar graph output of LimiTT, showing the number of miRNAs on the left and the number of MTIs on the right after specific processing steps of the pipeline.

b. MTI matrix

Within the MTI matrix file (***Table* 2**) all interactions between identified miRNAs and targets as UniProtAccs are marked by a binary string in the matrix, which represents the occurrence of the MTI over the chosen MTI DBs.

Table 2: MTI matrix output of LimiTT
Example of an MTI matrix output of LimiTT, which contains all identified MTIs between miRNAs (columns) and targets (rows: UniProtAccs). If an interaction between miRNA and target was identified, a binary number represents the occurrence of the interaction over the chosen MTI DBs. The order of the DBs for the binary string can be found in the first row.

Database order: TarBase, miRTarBase, miRecords, starBase							
	let-7b	miR-9	miR-15a	miR-17	miR-19b	miR-24	miR-26a
A0AVK6	0001				0110		
A2A6A1				1110			
A2AAY5			1110				1001
A2AHG0		0001	0101				

c. MTI information file

The MTI information file is a list of all identified target UniProtAccs combined with further information about these, collected during the process. Additional information consists of all identified interacting miRNAs, the original gene symbol within the MTI DBs, further gene synonyms, the target species, protein names, the UniProt review status, the Enzyme Commission number (EC number; [13]) and a list of Gene Ontology (GO; [14]) identifiers. If in the beginning of the process additional information from the annotation file and/or the miRNA list was specified, this information will also be part of the MTI information file.

d. MTI set overlap Heatmap

Based on the idea that each identified miRNA interacts with a set of target genes, the Heatmap (***Figure*** 4) depicts the ratio of overlapping UniProtAcc targets between each of these MTI sets. If the MTI set enrichment analysis was used, the Heatmap output will depict for each MTI set the ratio of overlapping target genes **which are part of the leading edge sets** of the corresponding MTI sets.

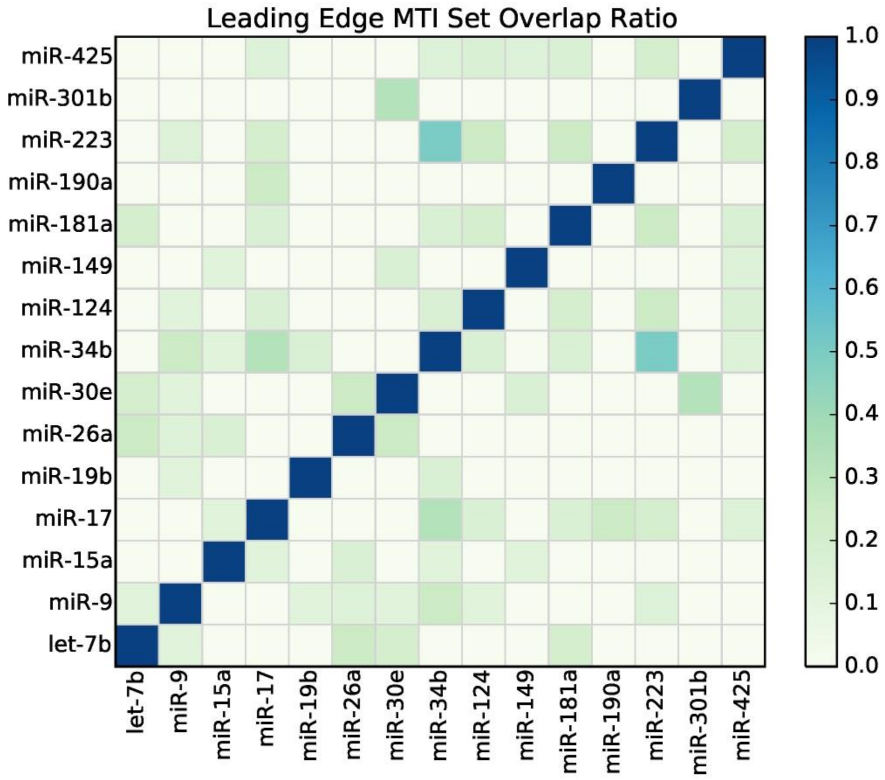

Figure 4: Heatmap output of LimiTT
Shown is an example of the symmetrical Heatmap output of LimiTT after the leading edge analysis. Depicted is the ratio of overlapping leading edge target genes for each identified miRNA interacting with target genes (MTI Set). The corresponding ratio is coloured based on the colour key on the right.

e. Ranked MTI sets file

After MTISEA, the ranking file contains the results of the analysis for each set of miRNA targets (***Table* 3**). For each MTI set, the size of the set is given, which is the number of overlapping UniProtAccs of the MTI set and the ranked list. Furthermore, the calculated ES, NES and FDR q-value are listed, together with the index of this element in the ranked file, for which the running sum statistic calculated the maximal ES. Additionally, the results of the leading edge analysis are given, which proceeds as follows: Depending on whether the ES of a MTI set is positive or negative, the set of leading edge targets either consists of the MTI set targets before or after the peak in the running sum calculation. Based on this, three statistics are calculated. "Tags" represents the ratio of leading edge targets to all targets in the given set. "List" calculates the ratio of UniProtAccs from the ranked dataset before/after the ES of the current set, to all UniProtAccs in the submitted file. "Signal" is a combination of the two

previous calculations, describing the distribution of the MTI set targets over the ranked dataset Thus, signal results in 100% or more, if all targets of the set can be found at the beginning of the ranked list.

Table 3: Ranked MTIs output of LimiTT
The table is an exemplary output of the ranked MTI sets of LimiTT. It contains the results of the MTI set enrichment analysis for each MTI set listed in column 1. Apart from the number of target genes in the set (column 2), the calculated Enrichment Score (ES; column 3), Normalized Enrichment Score (NES; column 4) and q-value of the False Discovery Rate (FDR q-val; column 5) are listed. The sixth column contains the rank within the ranking file at which the resulting ES was defined. Within column 9 are the three statistics tags, list and signal resulting from the Leading Edge analysis.

MTI Set	Size	ES	NES	FDR q-val	Rank at Max	Leading Edge
miR-149	6	0.65	1.55	0.290	16	tags=67%, list=29%, signal=53%
miR-301b	4	0.61	1.29	0.790	1	tags=25%, list=2%, signal=26%

If no MTI set enrichment analysis was started, the file contains the MTI sets ordered by the number of target genes within the sets.

f. Enrichment plots

Enrichments plots (***Figure* 5**) depict for each MTI set the running enrichments score over all UniProtAccs in the ranked dataset, the position of targets of the current MTI set within in the ranked list and the maximum ES, either positive or negative. Enrichment plots are created only if a MTI set enrichment analysis was started.

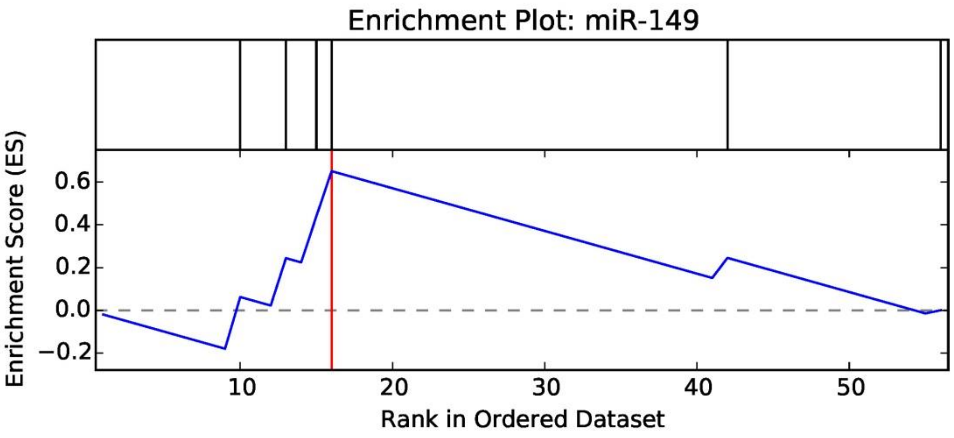

Figure 5: Enrichment Plot output of LimiTT
Depicted is the example of an Enrichment Plot for a MTI set named miR-149 from LimiTT with the running enrichment score for each of the UniProtAccs from the ranked list in blue, the positions of targets from the MTI set within the ranked dataset in black and the position of the maximum ES in red.

g. MTI set gene file

The MTI set gene file output of LimiTT is more or less a written version of all enrichment plots and thus just produced, if an enrichment analyses was initiated. The file lists for each MTI set, the targets which

overlap with the ranked list of UniProtAccs, the index of each of this targets within the ranked list, the running ES for this target and whether it is a member of the leading edge set or not (***Table* 4**).

Table 4: MTI set gene output of LimiTT

Exemplary MTI set gene file of LimiTT listing for all MTI sets in column 1: each target found within the ranked list in column 2, for each target the position of it in the ranked dataset (column 3), the running Enrichment Score (ES) and whether it is part of the Leading Edge (LE) set or not.

MTI set	Target	Index in Ranked List	Running ES	LE Member
miR-149	Q9WV91	10	0.06	Yes
miR-149	Q80SW1	13	0.24	Yes
miR-149	Q71B07	15	0.44	Yes
miR-149	A3KGS3	16	0.65	Yes
miR-149	Q80TA6	42	0.25	No
miR-149	Q69Z28	56	0.00	No
miR-181a	Q56A04	29	-0.16	Yes
miR-181a	A2AJK6	36	0.38	Yes
miR-190a	A3KGB4	14	0.76	Yes

6. Validation

Table 5: Top thirty published miRNA families and their members identified by LimiTT.
For each the number of validated targets identified by LimiTT is given in column three.

published miRNA Family	miRNA identified by LimiTT	Number of targets
miR-128/128ab	miR-128	88
	miR-128b	2
miR-130ac/301ab/301b/301b-3p/454/721/4295/3666	miR-130a	57
	miR-130b	71
	miR-301a	58
	miR-301b	59
	miR-3666	30
	miR-4295	35
	miR-454	57
miR-132/212/212-3p	miR-132	36
	miR-212	34
miR-135ab/135a-5p	miR-135a	45
	miR-135b	32
miR-139-5p	miR-139	43
miR-145	miR-145	34
miR-149	miR-149	29
miR-150/5127	miR-150	28
miR-153	miR-153	37
miR-155	miR-155	65
miR-15abc/16/16abc/195/322/424/497/1907	miR-15a	43
	miR-15b	45
	miR-16	65
	miR-195	44
	miR-424	44
	miR-497	47
miR-17/17-5p/20ab/20b-5p/93/106ab/427/518a-3p/519d	miR-106a	60
	miR-106b	64
	miR-17	77
	miR-20a	68
	miR-20b	66
	miR-518a	3
	miR-519d	58
	miR-93	68
miR-181abcd/4262	miR-181a	90
	miR-181b	94
	miR-181c	85
	miR-181d	91
	miR-4262	34
miR-182	miR-182	53
miR-1ab/206/613	miR-206	48
	miR-613	39
miR-200bc/429/548a	miR-200b	50

	miR-200c	50
	miR-429	54
miR-204/204b/211	miR-204	36
	miR-211	29
miR-218/218a	miR-218	41
miR-22	miR-22	29
miR-221/222/222ab/1928	miR-221	49
	miR-222	51
miR-23abc/23b-3p	miR-23a	83
	miR-23b	90
	miR-23c	24
miR-27ab	miR-27a	94
	miR-27b	97
miR-29abcd	miR-29a	32
	miR-29b	37
	miR-29c	30
miR-320abcd/4429	miR-320a	53
	miR-320b	37
	miR-320c	36
	miR-320d	36
	miR-4429	18
miR-33ab/33-5p	miR-33a	51
	miR-33b	40
miR-374ab	miR-374a	64
	miR-374b	61
miR-375	miR-375	29
miR-410/344de/344b-1-3p	miR-410	67
miR-495/1192	miR-495	108
miR-96/507/1271	miR-1271	43
	miR-507	3
	miR-96	48

Table 6: Top twenty-five MTI sets containing most highly connected genes.

The table contains the MTI sets (column 2) with the 25 highest normalised enrichment scores (NES; column 5) of all MTI sets interacting with the PH-related genes. For each MTI set its rank (column 1), miRNA family (column 3) and number of included genes (column 4) is given.

Rank	MTI Set	family	Size	NES
1	miR-432	mir-432	24	1.72
2	miR-302c	mir-302	40	1.71
3	miR-193b	mir-193	24	1.65
4	miR-28	mir-28	15	1.65
5	miR-302d	mir-302	35	1.61
6	miR-520b	mir-515	33	1.59
7	miR-4306		14	1.59
8	miR-193a	mir-193	10	1.58
9	miR-302e	mir-302_2	33	1.58
10	miR-185	mir-185	34	1.57

11	miR-302a	mir-302	35	1.57
12	miR-520e	mir-515	29	1.57
13	miR-520a	mir-515	34	1.57
14	miR-520c	mir-515	35	1.57
15	miR-155	mir-155	61	1.56
16	miR-302b	mir-302	35	1.56
17	miR-320b	mir-320	36	1.54
18	miR-708	mir-708	12	1.54
19	miR-376b	mir-368	41	1.53
20	miR-22	mir-22	28	1.52
21	miR-34a	mir-34	50	1.52
22	miR-376a	mir-368	38	1.52
23	miR-542	mir-542	25	1.52
24	miR-3619		10	1.52
25	mir-326	miR-326	27	1.51

7. References

1. Vergoulis T, Vlachos IS, Alexiou P, Georgakilas G, Maragkakis M, Reczko M et al. TarBase 6.0: capturing the exponential growth of miRNA targets with experimental support. Nucleic acids research. 2012;40(Database issue):D222-9. doi:10.1093/nar/gkr1161.
2. Hsu SD, Tseng YT, Shrestha S, Lin YL, Khaleel A, Chou CH et al. miRTarBase update 2014: an information resource for experimentally validated miRNA-target interactions. Nucleic acids research. 2014;42(Database issue):D78-85. doi:10.1093/nar/gkt1266.
3. Xiao F, Zuo Z, Cai G, Kang S, Gao X, Li T. miRecords: an integrated resource for microRNA-target interactions. Nucleic acids research. 2009;37(Database issue):D105-10. doi:10.1093/nar/gkn851.
4. Li JH, Liu S, Zhou H, Qu LH, Yang JH. starBase v2.0: decoding miRNA-ceRNA, miRNA-ncRNA and protein-RNA interaction networks from large-scale CLIP-Seq data. Nucleic acids research. 2014;42(Database issue):D92-7. doi:10.1093/nar/gkt1248.
5. Griffiths-Jones S, Grocock RJ, van Dongen S, Bateman A, Enright AJ. miRBase: microRNA sequences, targets and gene nomenclature. Nucleic acids research. 2006;34(Database issue):D140-4. doi:10.1093/nar/gkj112.
6. Magrane M, Consortium U. UniProt Knowledgebase: a hub of integrated protein data. Database : the journal of biological databases and curation. 2011;2011:bar009. doi:10.1093/database/bar009.
7. Pruitt K, Brown G, Tatusova T, Maglott D. The Reference Sequence (RefSeq) Database. In: McEntyre J, Ostell J, editors. The NCBI Handbook [Internet]. Bethesda (MD): National Center for Biotechnology Information (US)2002 [Updated 2012].
8. Pontius JU, Wagner L, Schuler GD. UniGene: A Unified View of the Transcriptome. In: McEntyre J, Ostell J, editors. The NCBI Handbook [Internet]. Bethesda (MD): National Center for Biotechnology Information (US)2002.
9. Cunningham F, Amode MR, Barrell D, Beal K, Billis K, Brent S et al. Ensembl 2015. Nucleic acids research. 2015;43(Database issue):D662-9. doi:10.1093/nar/gku1010.
10. Blanco E, Parra G, Guigo R. Using geneid to identify genes. Current protocols in bioinformatics / editoral board, Andreas D Baxevanis [et al]. 2007;Chapter 4:Unit 4 3. doi:10.1002/0471250953.bi0403s18.
11. Kanehisa M, Goto S. KEGG: kyoto encyclopedia of genes and genomes. Nucleic acids research. 2000;28(1):27-30.
12. Kuenne C, Preussner J, Herzog M, Braun T, Looso M. MIRPIPE: quantification of microRNAs in niche model organisms. Bioinformatics. 2014;30(23):3412-3. doi:10.1093/bioinformatics/btu573.
13. Webb EC. Enzyme nomenclature 1992. Recommendations of the Nomenclature Committee of the International Union of Biochemistry and Molecular Biology on the Nomenclature and Classification of Enzymes. vol Ed. 6. Academic Press; 1992.
14. Ashburner M, Ball CA, Blake JA, Botstein D, Butler H, Cherry JM et al. Gene Ontology: tool for the unification of biology. Nature genetics. 2000;25(1):25-9.

8. Manual

Contents

Contents

HowTo: Load example data.......... 17

HowTo: miRNA identification.......... 18

HowTo: miRNA enrichment analysis.......... 19

HowTo: miRNA target search.......... 20

HowTo: miRNA target identification in respect to an gene profile.......... 21

HowTo: miRNA target identification in respect to an expression profile.......... 22

HowTo: Inspect and download result tables/figures.......... 23

Test data details are illustrated in the paper Bayer et al. (2016), the dataset is derived from pulmonary hypertension disease, Bertero et al. (2014).

HowTo: Load example data

1. Browse to the shared data library to load example files for LimiTT into galaxy.

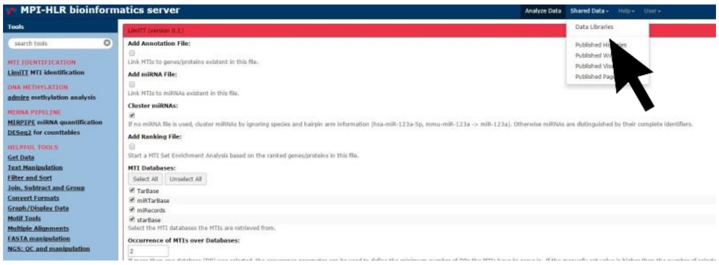

2. The given annotation file was used to benchmark LimiTT and it contains PH-relevant genes from Bertero et al. (2014). To reproduce the benchmarking, select all the files given in the example library and import them to your history by clicking “Go”.

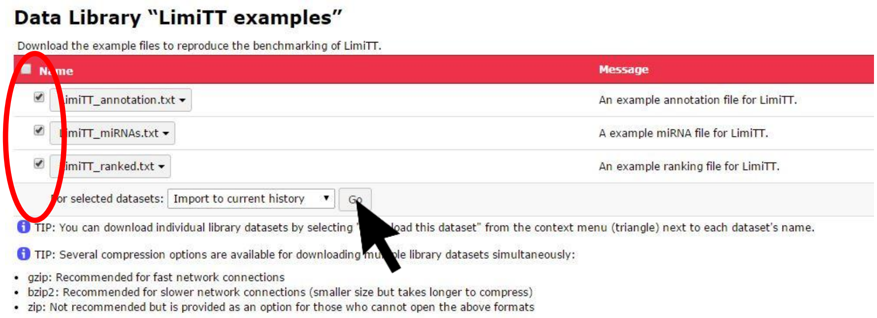

3. The example files will appear in your personal history

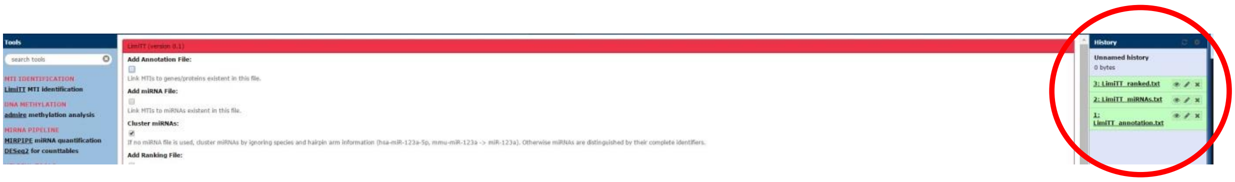

HowTo: miRNA identification

1. Select the LimiTT tool, click on "Add annotation file" and use the LimiTT_annotation.txt as input into the pipeline. Next, change the "Description of additional information" field from "Transcript" to "Functional Pathway". Change the "Occurrence of MTIs over DBs" value to one. Select "Homo sapiens" as species. Hit "Execute" to see the tool in action.

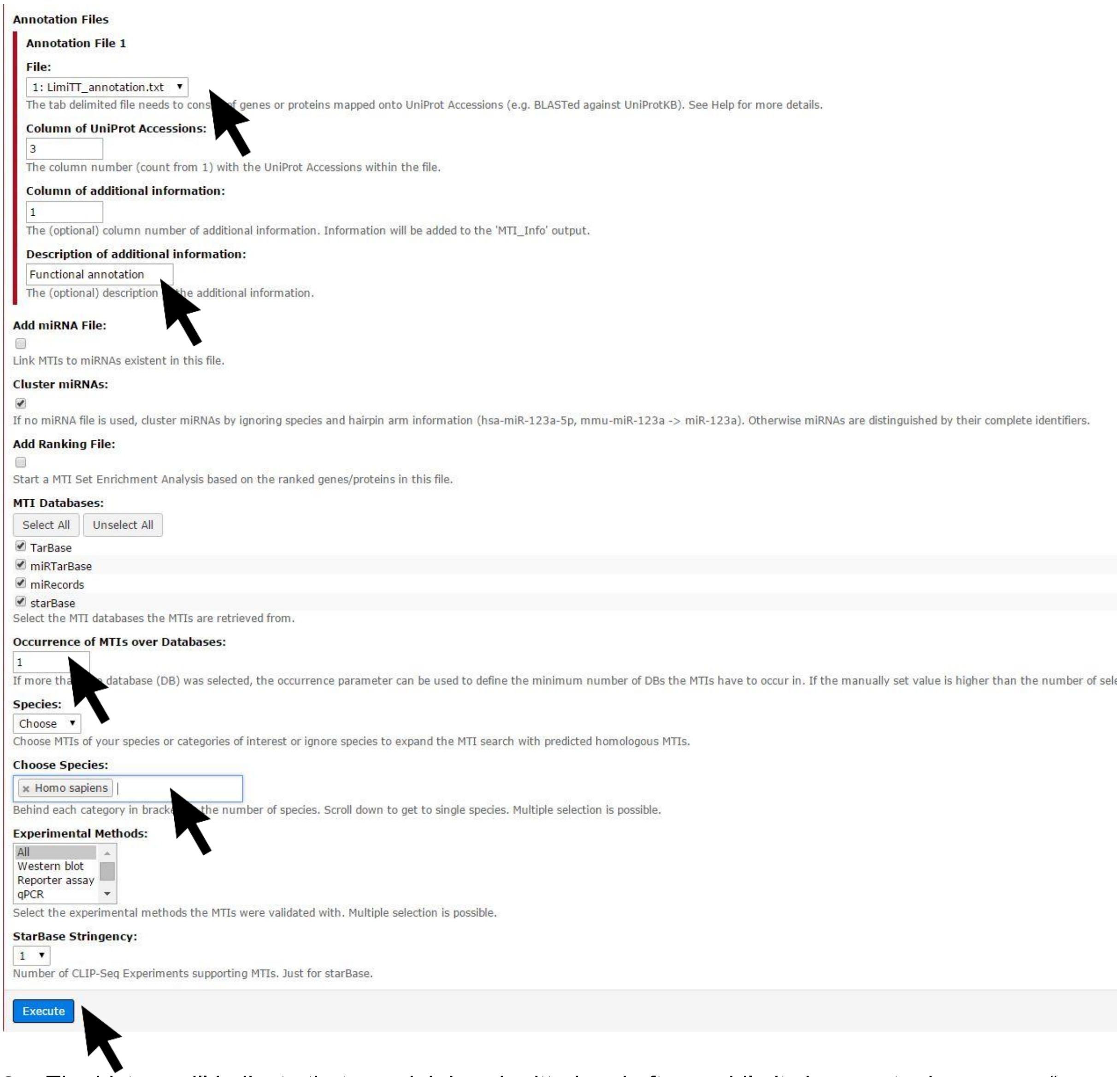

2. The history will indicate that your job is submitted and after a while it changes to „in progress“ (yellow).

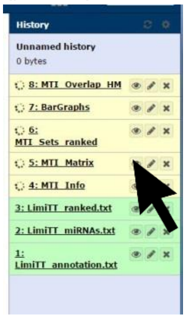

HowTo: miRNA enrichment analysis

1. Select the LimiTT tool, click on "Add ranking file" and use the LimiTT_ranked.txt as input into the pipeline. Next, change the "Occurrence of MTIs over DBs" value to one. Select "Homo sapiens" as species. Hit "Execute" to see the tool in action.

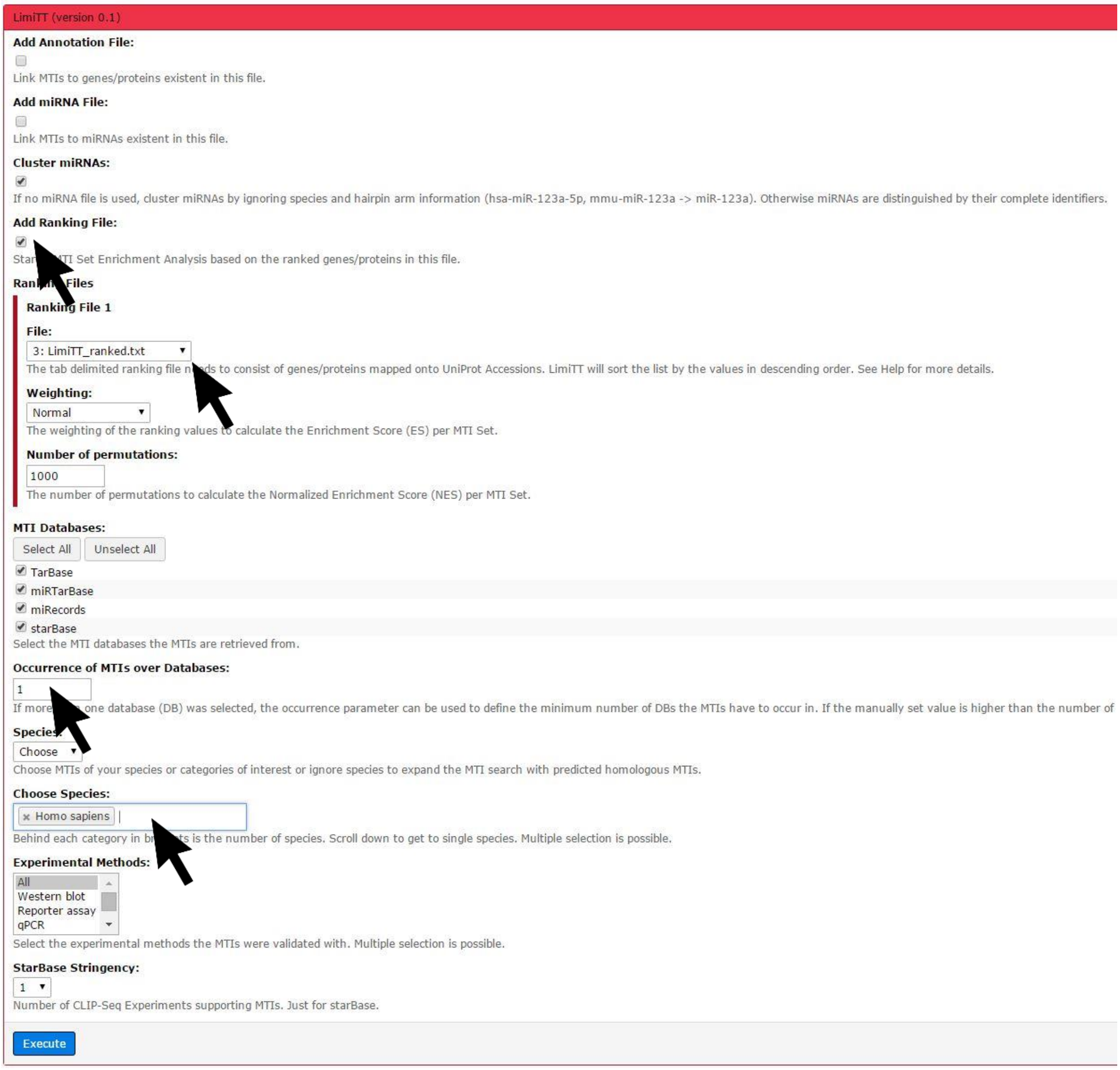

2. The history will indicate that your job is submitted and after a while it changes to „in progress“ (yellow). The set of result files generated is different from the set generated in use case 1.

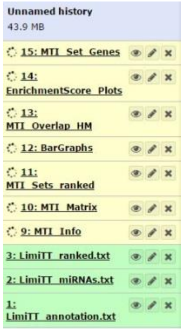

HowTo: miRNA target search

1. Select the LimiTT tool, click on "Add miRNA file" and use the LimiTT_miRNAs.txt as input into the pipeline. Next, change the "Occurrence of MTIs over DBs" value to one. Select "Homo sapiens" as species. Hit "Execute" to see the tool in action. If you want to search for other species or a combined list of species, choose/add for instance *animal* and/or *mus musculus.*

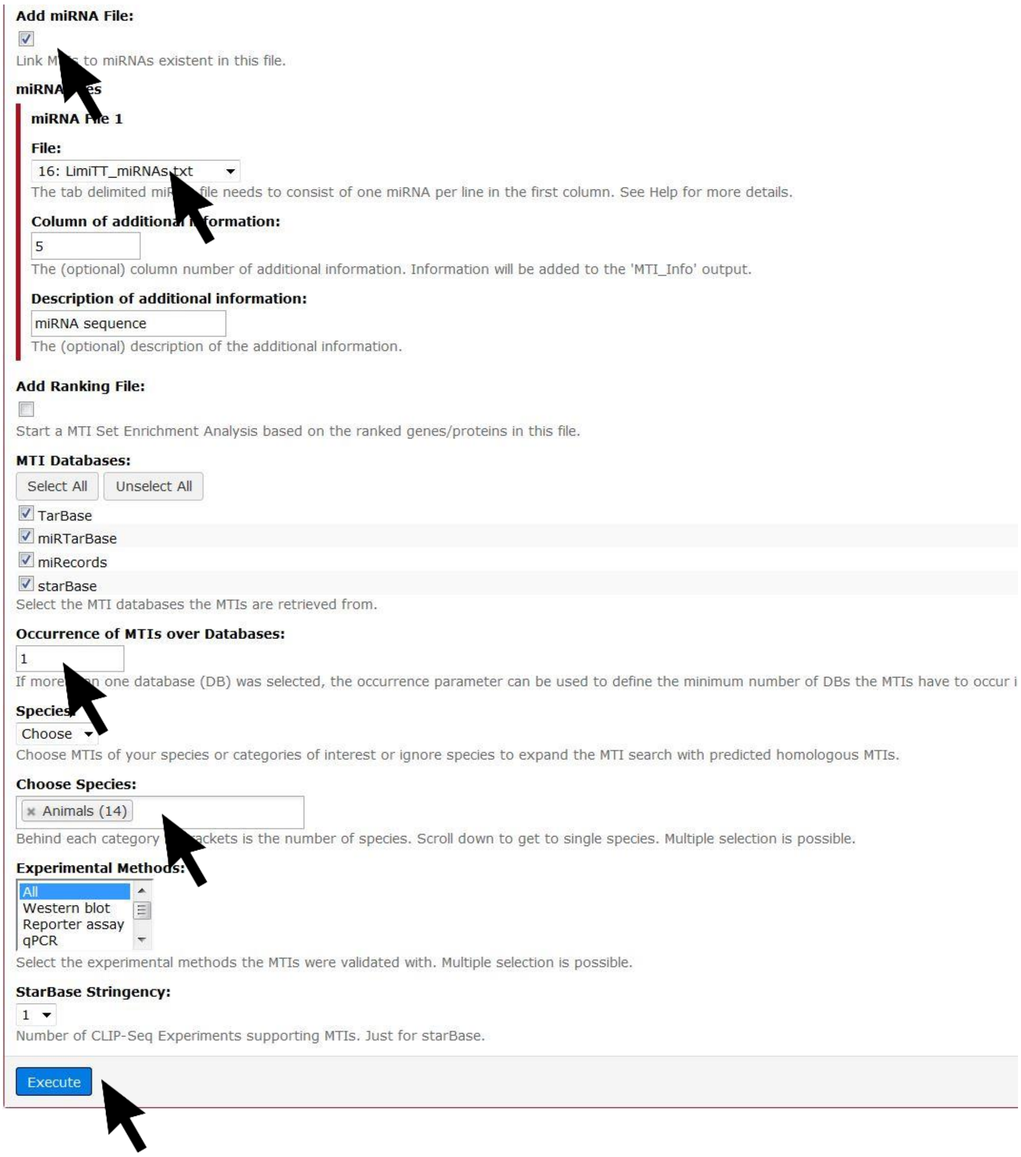

2. The history will indicate that your job is submitted and after a while it changes to „in progress“ (yellow). The set of result files generated is different from the set generated in use case 1.

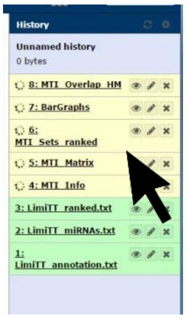

HowTo: miRNA target identification in respect to an gene profile

1. Select the LimiTT tool, click on "Add miRNA file" and use the LimiTT_miRNAs.txt as input into the pipeline. Next, click on "Add annotation file" and use the LimiTT_annotations.txt as input into the pipeline. Select "Homo sapiens" as species. Hit "Execute" to see the tool in action.

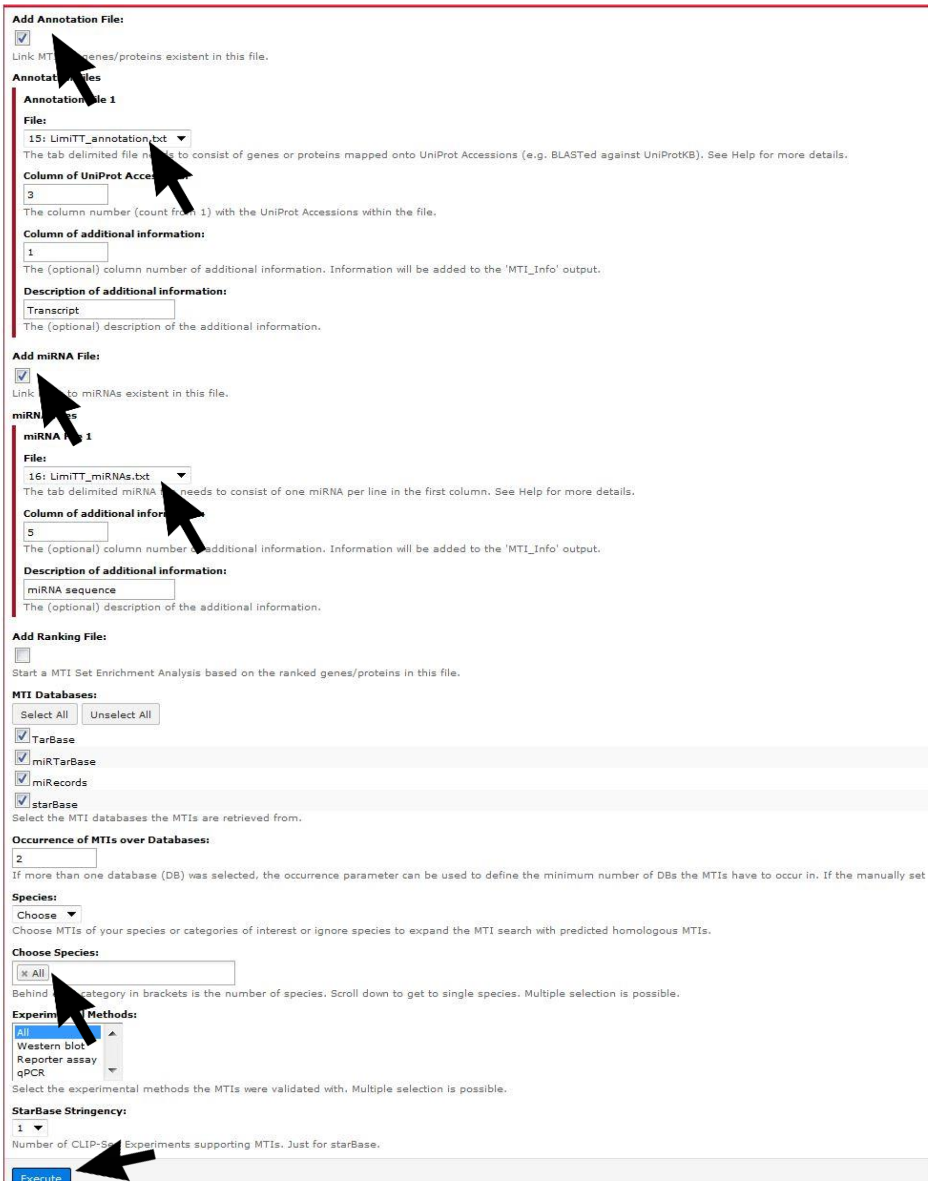

2. The history will indicate that your job is submitted and after a while it changes to „in progress“ (yellow). The set of result files generated is different from the set generated in use case 1.

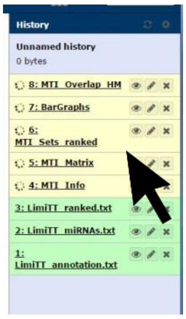

HowTo: miRNA target identification in respect to an expression profile

1. Select the LimiTT tool, click on "Add miRNA file" and use the LimiTT_miRNAs.txt as input into the pipeline. Next, click on "Add ranking file" and use the LimiTT_ranking.txt as input into the pipeline. Next, click on "Add annotation file" and use the LimiTT_annotations.txt as input into the pipeline (might be the total gene list). Select "Homo sapiens" as species. Hit "Execute" to see the tool in action.

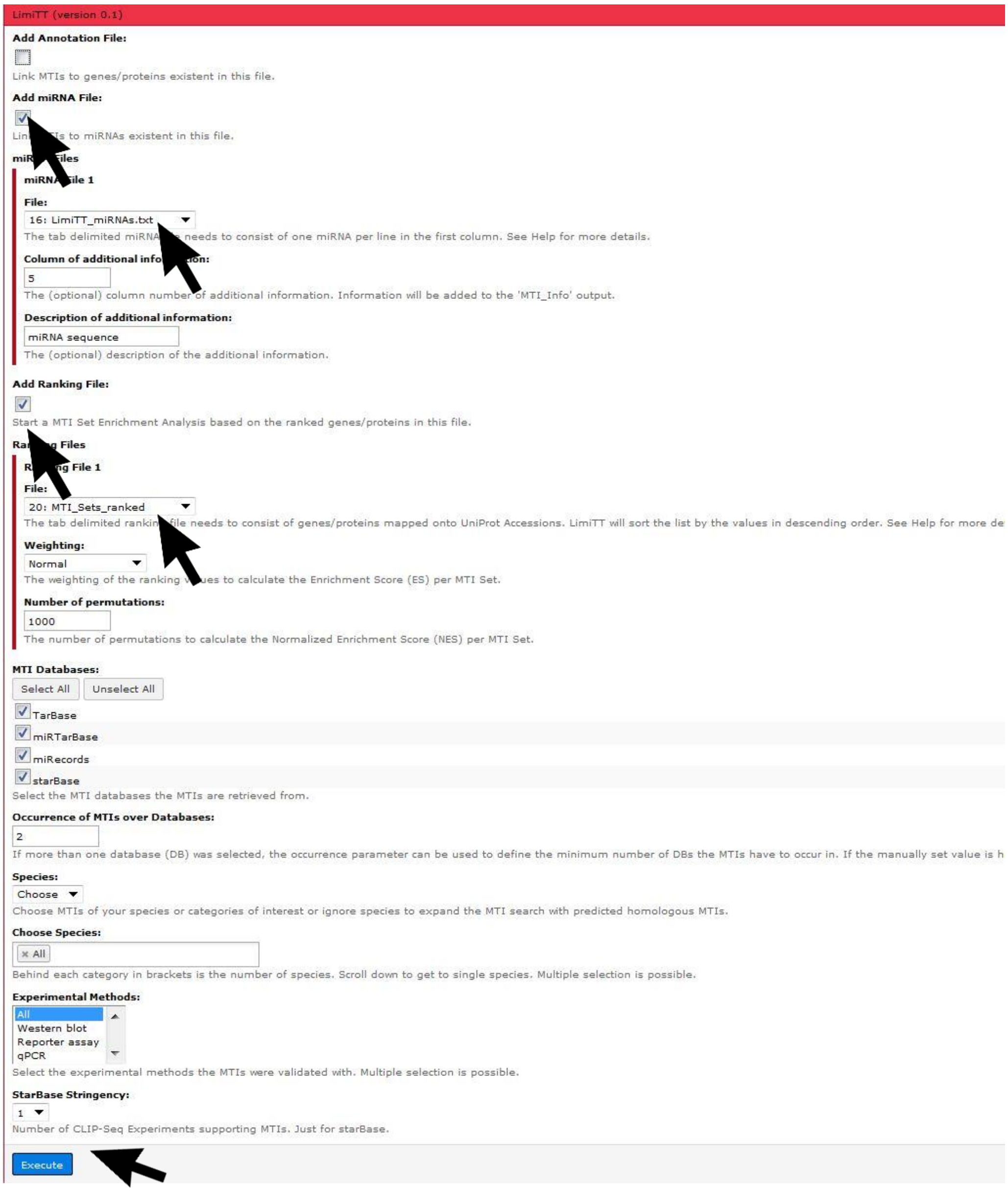

2. The history will indicate that your job is submitted and after a while it changes to „in progress“ (yellow). The set of result files generated is different from the set generated in use case 1.

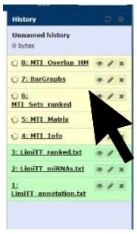

HowTo: Inspect and download result tables/figures

1. Once the analysis is done (might take some hours in respect to workload on the server and length of ID lists transferred, we do not limit the size of the lists !!), the result files change to green.

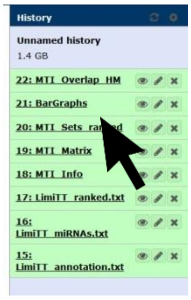

2. The resulting files in the history tab can be visualized by clicking the *EYE* symbol. For further offline processing, the files can be downloaded by the *SAVE* symbol.

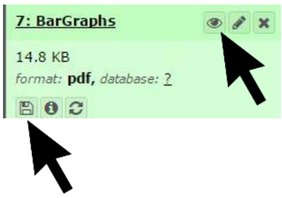

Supplement: Additional file 1: — Data: Supplementary figures and descriptions helpful for the understanding of LimiTT. (PDF 1745 kb) [file 12859_2016_1070_MOESM1_ESM.pdf]
